# Supplementary material for: A Global Screen for Assembly State Changes of the Mitotic Proteome by SEC-SWATH-MS
Source: Cell Syst. 2020 Feb 26;10(2):133–155.e6. doi: 10.1016/j.cels.2020.01.001 (PMC7042714; doi:10.1016/j.cels.2020.01.001)

Q96CV9 | OPTN_HUMAN | OPTN FIP2 GLC1E HIP7 HYPL NRP
Monomer MW [kDa]: 65.921 Monomer expected elution fraction: 44

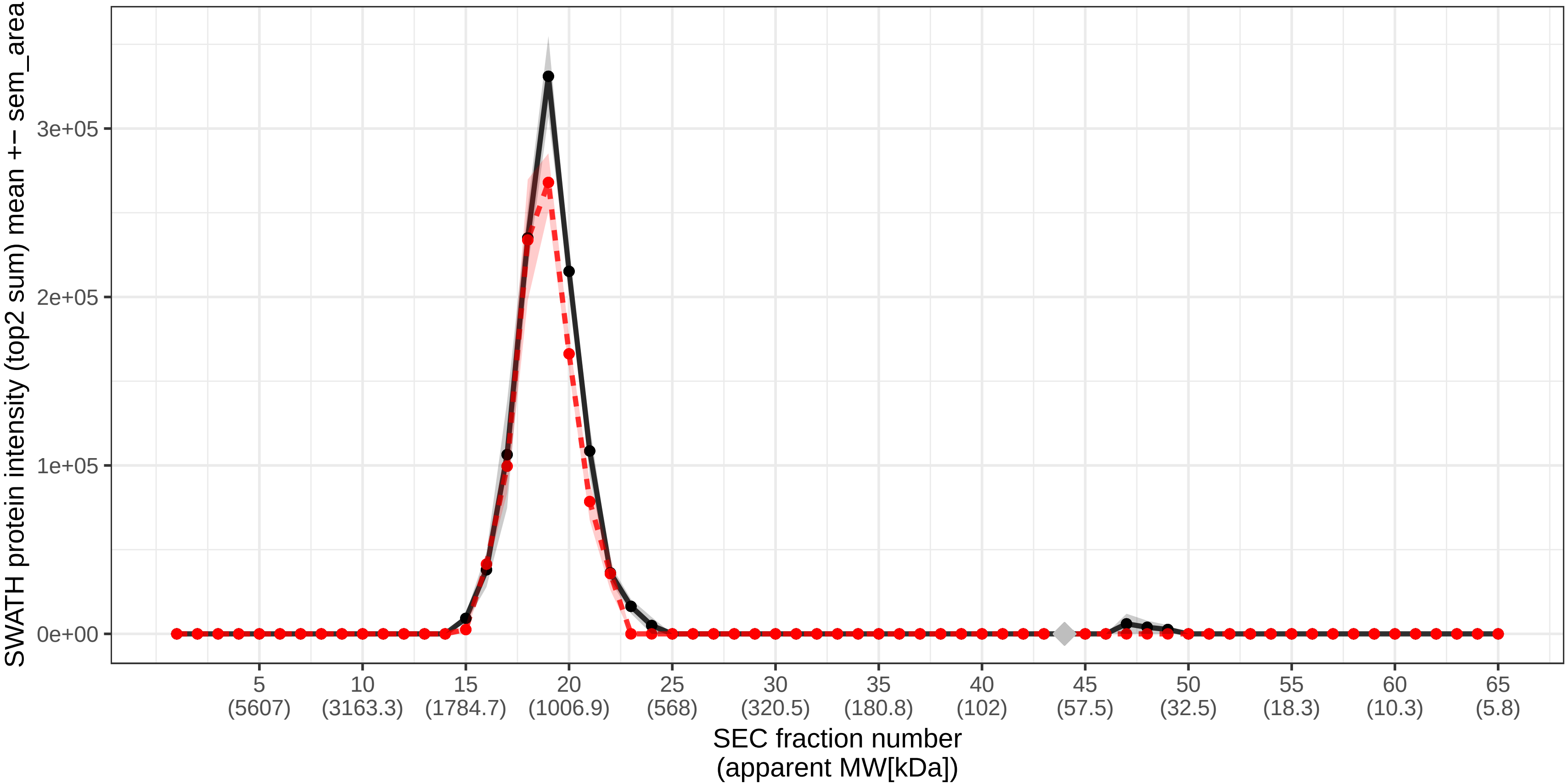

Supplement: Data S1. SEC-SWATH-MS Protein Chromatograms, Related to Figure 1 [file mmc6.zip › SECchrom_Q96CV9_OPTN_HUMAN_OPTN_FIP2_GLC1E_HIP7_HYPL_NRP.pdf]
